# Association of ANA and SSA autoantibodies with progression-free survival in multiple myeloma: a retrospective cohort study

**DOI:** 10.3389/fonc.2025.1529678

**Published:** 2025-02-21

**Authors:** Jiafeng Zhang, Hefei Ren, Lei Chen, Xin Wang, Huiquan Wang, Hongkun Wu, Lin Zhou

**Affiliations:** Department of Laboratory Medicine, Shanghai Changzheng Hospital, Naval Medical University, Shanghai, China

**Keywords:** multiple myeloma, autoantibodies, ANA (antinuclear antibody), SSA (Anti-Sjögren’s-syndrome-related Antigen A), progression-free survival, propensity score matching (PSM)

## Abstract

**Objective:**

This study aimed to investigate the relationship between autoantibodies, specifically Antinuclear Antibody (ANA) and anti-Sjögren’s-syndrome-related antigen A (SSA), and progression-free survival (PFS) in multiple myeloma (MM) patients.

**Methods:**

A retrospective cohort study was conducted on 304 MM patients diagnosed between 2010 and 2020 at Shanghai Changzheng Hospital, with follow-up until October 2023. Patients were stratified based on ANA and SSA positivity. Clinical data were analyzed using Kaplan-Meier survival curves and Cox regression models, adjusting for key prognostic factors. Sensitivity analyses were performed using propensity score matching (PSM) and inverse probability of treatment weighting (IPTW) to evaluate the robustness of the results.

**Results:**

SSA-positive patients exhibited significantly shorter PFS compared to SSA-negative patients (17 vs. 44 months, HR = 2.93, 95% CI 1.53-5.64, p = 0.001), while ANA positivity was associated with a smaller increase in risk (HR = 1.57, 95% CI 1.04-2.4, p = 0.034). The impact of SSA remained significant after adjusting for various covariates in the Cox regression model and sensitivity analyses. Subgroup analyses revealed consistent effects of SSA positivity across different demographic and clinical factors.

**Conclusion:**

SSA positivity is associated with a higher risk of disease progression in MM patients, suggesting it may serve as a valuable prognostic marker. The relationship between autoantibodies and MM prognosis warrants further investigation in larger, multicenter studies to elucidate the underlying mechanisms and inform clinical management.

## Introduction

Multiple myeloma (MM) is a malignancy characterized by the clonal proliferation of abnormal plasma cells in the bone marrow (1). While numerous studies have explored the association between autoimmune diseases and the risk of MM development, the specific impact of autoantibodies on MM prognosis remains unclear (2–7). Given the immunological basis of both autoimmune diseases and MM, understanding the role of autoantibodies could have significant implications for disease management and patient outcomes (8).

Previous research has demonstrated a link between a history of autoimmune diseases and an increased risk of MM (3–7, 9, 10). Additionally, the presence of specific autoantibodies, such as anti-Sjögren’s-syndrome-related antigen A (SSA) and anti-Sjögren’s-syndrome-related antigen B, or anti-La antibodies (SSB) has been associated with an elevated risk of MM in certain autoimmune conditions, such as Sjögren’s syndrome (11). These findings raise important questions about the potential prognostic value of commonly used diagnostic markers for autoimmune diseases—autoantibodies—in the context of MM.

This study aims to address this gap by investigating the relationship between autoantibody profiles and progression-free survival (PFS) in patients with MM, with a particular focus on Antinuclear Antibody (ANA), and SSA. By assessing the influence of autoantibody status on PFS, we aim to provide new insights into the role of immune dysregulation in the prognosis of MM.

## Methods

### Study population

Patients diagnosed with MM at Shanghai Changzheng Hospital between January 1, 2010, and January 1, 2020, were included in this cohort study, with follow-up continuing until October 2023. Patients with monoclonal gammopathy of undetermined significance (MGUS), solitary plasmacytoma, or smoldering multiple myeloma (SMM) were excluded. The study received approval from the Clinical Ethics Committee of Shanghai Changzheng Hospital (approval number: 2023SLYS7) prior to its initiation. Informed consent was obtained from all participants prior to their inclusion in the study. A unique study ID was assigned to each patient to ensure data anonymization.

### Measurements and covariates

Data for this cohort study were collected using the Hospital Information System (HIS) and Laboratory Information System (LIS) at Shanghai Changzheng Hospital, offering comprehensive access to demographic, clinical, laboratory, and treatment information for all MM patients.

The primary laboratory and clinical data were gathered at the time of MM diagnosis, with missing data supplemented by records from within two months before or after the diagnosis. If data within this timeframe were unavailable, those laboratory values were treated as missing and excluded from the analysis. All laboratory data were collected prior to the initiation of the first chemotherapy.

Covariates were chosen based on known or potential impacts on MM progression and treatment outcomes, including age, sex, M protein type, light chain restriction, hemoglobin (Hb) level, serum β2-microglobulin (β2M), lactate dehydrogenase (LDH), serum calcium (Ca), and bone marrow plasma cells (BMPC), and the free light chain ratio (FLCR). High-risk cytogenetic abnormalities were defined per the Mayo myeloma stratification system, including t(4;14), t(14;16), t(14;20), del(17p), and 1q gains (12). Disease classification was based on the Durie-Salmon (DS) stage, International Staging System (ISS), and Revised ISS (R-ISS) (13–15). Additional variables included autologous stem cell transplantation (ASCT) status, the use of proteasome inhibitors (bortezomib, carfilzomib, ixazomib), immunomodulators (lenalidomide, thalidomide, pomalidomide), and monoclonal antibodies (daratumumab, isatuximab, elotuzumab, belantamab mafodotin). Renal disease, hypertension (16), diabetes (17), and autoimmune diseases were considered based on medical history.

Renal disease was defined as a documented history of chronic kidney disease (CKD), acute kidney injury (AKI), or any form of renal impairment, as diagnosed by a nephrologist (18). Autoimmune diseases were defined as a diagnosis of conditions such as systemic lupus erythematosus (SLE), rheumatoid arthritis (RA), Sjögren’s syndrome, or other autoimmune disorders based on established diagnostic criteria and confirmed by clinical records (19–21).

All data on patient comorbidities and clinical characteristics were extracted from electronic medical records. Staging classifications were determined based on international guidelines, with each patient’s staging independently evaluated by two experienced hematology clinicians and subsequently reviewed by a chief hematologist for accuracy.

### Autoantibody profiles

Autoantibody results obtained during the study via LIS included ANAs and SSA, which were detected using the indirect immunofluorescence (IIF) method (Euroimmun, Lubeck, Germany) and the Euroline ANA Profile (Euroimmun, Lubeck, Germany), following the manufacturer’s protocols (**Supplementary Figure S1**).

For ANA detection, we adopted a cutoff titer of ≥1:160, based on the International Consensus on ANA Patterns (ICAP) guidelines and American College of Rheumatology (ACR) recommendations (22).

For SSA detection, we used a semi-quantitative immunoblot assay, following the manufacturer’s classification criteria. Results were categorized as positive, negative, or borderline, and only definitively positive results were considered SSA-positive to ensure clinical relevance and reduce potential misclassification bias.

To ensure test reliability, all laboratory analyses were conducted in a single certified laboratory with strict internal quality controls, and all assays met the manufacturer’s specified quality criteria. Additionally, all positive ANA and SSA results were reviewed by experienced clinicians to verify their clinical relevance in the study context.

Autoantibody screening was performed at any time before the patient was diagnosed with MM or before the initiation of treatment following diagnosis.

### Treatment response assessment

In accordance with the International Myeloma Working Group (IMWG) criteria for treatment response, treatment response was evaluated after initial induction therapy (23). Patients were categorized into the following response groups: stringent complete response (sCR), complete response (CR), very good partial response (VGPR), partial response (PR), stable disease (SD), and progressive disease (PD). These response classifications were extracted from electronic medical records and cross-validated by two independent hematologists to ensure accuracy.

### Statistical analyses

Baseline characteristics were compared between patients with different autoantibody results using chi-square tests for categorical variables and independent samples t-tests or Mann-Whitney U tests for continuous variables, depending on normality. A two-tailed significance level of α = 0.05 was used for all analyses. All analyses were performed using the statistical software packages R (http://www.R-project.org, The R Foundation and d Free Statistics software versions 1.6).

The primary objective of this study was to evaluate the relationship between autoantibody and prognosis in MM patients. To achieve this, we utilized Cox regression analysis, controlling for several important baseline MM prognostic factors including age, gender, DS stage, ISS stage, R-ISS stage, MM treatment, and comorbidities. An extended Cox model approach was applied to adjust the model for various covariates. Survival curves were plotted using Kaplan-Meier and log-rank analyses. Subgroup analyses were conducted, stratified by a number of relevant effect covariates.

In the sensitivity analysis, we employed both propensity score matching (PSM) and an inverse probability of treatment weighting (IPTW) model to assess the robustness of our results. PSM was conducted using a 1:1 nearest neighbor matching algorithm with a caliper width of 0.2, while IPTW was truncated at the 5th and 95th percentiles (24, 25). All covariates were included in the generation of the propensity scores. The quality of the PSM was evaluated using the standardized mean difference (SMD), with a threshold of less than 0.1 considered acceptable, indicating a well-balanced distribution of covariates between the matched groups.

## Result

### Autoantibody selection

In this study, we initially screened a comprehensive panel of autoantibodies, including ANA,SSA, SSB, anti-topoisomerase I (SCL-70), anti-histidyl-tRNA synthetase (Jo-1), anti-histone antibody (AHA), anti-U1 ribonucleoprotein (U1-RNP), anti-nucleolar antibody (ANuA), anti-RNA antibody (ARA), and anti-Smith antibody. Among the 304 patients, 51 (16.8%) were ANA-positive and 13 (4.3%) were SSA-positive. The remaining autoantibodies showed very low positivity rates (**Supplementary Figure S2**). Due to their low prevalence, only ANA and SSA were included in the final analysis, as statistical power to detect meaningful associations was limited (24).

### Study population characteristics

**Table 1** summarizes the baseline characteristics of the study population, stratified by ANA and SSA status. A total of 304 patients were enrolled, with a mean age of 60 years. Among them, 170 were male (55.9%) and 134 were female (44.1%). The most common M protein type was IgG (48%). 14 patients were diagnosed with autoimmune diseases, within 8 ANA-positive and 2 SSA-positive. Baseline characteristics and results in 14 MM patients diagnosed with autoimmune disease are presented in **Supplementary Table S1**. Statistically significant differences (p < 0.05) were observed in the ANA group for sex, M protein type, hemoglobin level, serum β2-microglobulin, serum creatinine, ISS stage, R-ISS stage, and autoimmune disease. In the SSA group, only hemoglobin level showed a significant difference.

**Table 1:** Baseline characteristics of the study population.

Characteristics	Total (n=304)	ANA		SSA	
		Negative (n=253)	Positive (n=51)	Negative (n=291)	Positive (n=13)
Age, mean (SD), years	60.0 (11.9)	59.5 (11.8)	62.3 (12.0)	60.0 (11.9)	59.6 (11.1)
Age at diagnosis, n (%)					
<65	179 (58.9)	154 (60.9)	25 (49)	171 (58.8)	8 (61.5)
≥65	125 (41.1)	99 (39.1)	26 (51)	120 (41.2)	5 (38.5)
Sex, n(%)			*		
Female	134 (44.1)	118 (46.6)	16 (31.4)	130 (44.7)	4 (30.8)
Male	170 (55.9)	135 (53.4)	35 (68.6)	161 (55.3)	9 (69.2)
M protein type, n (%)			*		
Ig G	146 (48.0)	124 (49)	22 (43.1)	140 (48.1)	6 (46.2)
Ig A	75 (24.7)	63 (24.9)	12 (23.5)	73 (25.1)	2 (15.4)
Ig D	28 (9.2)	26 (10.3)	2 (3.9)	26 (8.9)	2 (15.4)
Light chain only	36 (11.8)	22 (8.7)	14 (27.5)	33 (11.3)	3 (23.1)
Others	19 (6.2)	18 (7.1)	1 (2)	19 (6.5)	0 (0)
Light chain restriction, n (%)					
Kappa	153 (50.3)	126 (49.8)	27 (52.9)	148 (50.9)	5 (38.5)
Lambdad	151 (49.7)	127 (50.2)	24 (47.1)	143 (49.1)	8 (61.5)
Hemoglobin level, n (%)			*		*
<100	164 (53.9)	129 (51)	35 (68.6)	153 (52.6)	11 (84.6)
≥100	140 (46.1)	124 (49)	16 (31.4)	138 (47.4)	2 (15.4)
Serum β2-microglobulin, n (%)			*		
<3.5	127 (41.8)	114 (45.1)	13 (25.5)	123 (42.3)	4 (30.8)
≥3.5	177 (58.2)	139 (54.9)	38 (74.5)	168 (57.7)	9 (69.2)
Serum LDH, n (%)					
<245	160 (52.6)	132 (52.2)	28 (54.9)	152 (52.2)	8 (61.5)
≥245	144 (47.4)	121 (47.8)	23 (45.1)	139 (47.8)	5 (38.5)
Serum calcium level, n (%)					
<2.65	282 (92.8)	237 (93.7)	45 (88.2)	269 (92.4)	13 (100)
≥2.65	22 (7.2)	16 (6.3)	6 (11.8)	22 (7.6)	0 (0)
Serum creatinine level, n (%)			*		
<177	263 (86.5)	225 (88.9)	38 (74.5)	253 (86.9)	10 (76.9)
≥177	41 (13.5)	28 (11.1)	13 (25.5)	38 (13.1)	3 (23.1)
Plasma cells of BM, n (%)^a^					
<30	157 (51.6)	127 (50.2)	30 (58.8)	150 (51.5)	7 (53.8)
≥30	147 (48.4)	126 (49.8)	21 (41.2)	141 (48.5)	6 (46.2)
High risk cytogenetic abnormalitie, n (%)^b^					
No	64 (21.1)	56 (22.1)	8 (15.7)	62 (21.3)	2 (15.4)
Yes	240 (78.9)	197 (77.9)	43 (84.3)	229 (78.7)	11 (84.6)
DS stage, n (%)					
I	26 (8.6)	22 (8.7)	4 (7.8)	26 (8.9)	0 (0)
II	24 (7.9)	19 (7.5)	5 (9.8)	23 (7.9)	1 (7.7)
III	254 (83.6)	212 (83.8)	42 (82.4)	242 (83.2)	12 (92.3)
ISS stage, n (%)			*		
I	68 (22.4)	62 (24.5)	6 (11.8)	66 (22.7)	2 (15.4)
II	100 (32.9)	90 (35.6)	10 (19.6)	97 (33.3)	3 (23.1)
III	136 (44.7)	101 (39.9)	35 (68.6)	128 (44)	8 (61.5)
R-ISS stage, n (%)			*		
I	35 (11.5)	33 (13)	2 (3.9)	33 (11.3)	2 (15.4)
II	178 (58.6)	152 (60.1)	26 (51)	172 (59.1)	6 (46.2)
III	91 (29.9)	68 (26.9)	23 (45.1)	86 (29.6)	5 (38.5)
ASCT, n (%)					
No	240 (78.9)	196 (77.5)	44 (86.3)	228 (78.4)	12 (92.3)
Yes	64 (21.1)	57 (22.5)	7 (13.7)	63 (21.6)	1 (7.7)
Proteasome inhibitor, n (%)					
No	55 (18.1)	48 (19)	7 (13.7)	52 (17.9)	3 (23.1)
Yes	249 (81.9)	205 (81)	44 (86.3)	239 (82.1)	10 (76.9)
Immunomodulator, n (%)					
No	119 (39.1)	102 (40.3)	17 (33.3)	115 (39.5)	4 (30.8)
Yes	185 (60.9)	151 (59.7)	34 (66.7)	176 (60.5)	9 (69.2)
Monoclonal Antibodies, n (%)^c^					
No	280 (92.1)	235 (92.9)	45 (88.2)	267 (91.8)	13 (100)
Yes	24 (7.9)	18 (7.1)	6 (11.8)	24 (8.2)	0 (0)
Renal disease, n (%)^d^					
No	275 (90.5)	227 (89.7)	48 (94.1)	264 (90.7)	11 (84.6)
Yes	29 (9.5)	26 (10.3)	3 (5.9)	27 (9.3)	2 (15.4)
Hypertension, n (%)					
No	189 (62.2)	156 (61.7)	33 (64.7)	178 (61.2)	11 (84.6)
Yes	115 (37.8)	97 (38.3)	18 (35.3)	113 (38.8)	2 (15.4)
Diabetes, n (%)					
No	265 (87.2)	218 (86.2)	47 (92.2)	252 (86.6)	13 (100)
Yes	39 (12.8)	35 (13.8)	4 (7.8)	39 (13.4)	0 (0)
Autoimmune disease, n (%)^e^			*		
No	290 (95.4)	247 (97.6)	43 (84.3)	279 (95.9)	11 (84.6)
Yes	14 (4.6)	6 (2.4)	8 (15.7)	12 (4.1)	2 (15.4)

Ig G, Immunoglobulin G; Ig A, Immunoglobulin A; Ig D, Immunoglobulin D; LDH, Lactate Dehydrogenase; BM, Bone Marrow; DS stage, Durie-Salmon Staging System; ISS stage, International Staging System; R-ISS stage, Revised International Staging System; ASCT, Autologous Stem Cell Transplant; ANA, Antinuclear Antibody; SSA, Anti-Sjögren’s-Syndrome-Related Antigen A.

* indicates a statistically significant difference between the two groups with a p-value less than 0.05; ^a^The percentage of plasma cells in bone marrow at initial diagnosis, determined by bone marrow aspiration; ^b^High-risk cytogenetic abnormalities are defined by the Mayo myeloma risk stratification system and include t(4;14), t(14;16), t(14;20), del(17p), and 1q gains. All other types are considered non-high-risk cytogenetic abnormalities; ^c^Refers to the use of targeted antibody therapies in treatment, such as Daratumumab, Isatuximab, Elotuzumab, or Belantamab mafodotin, administered to patients as part of their multiple myeloma therapy regimen; ^d^Renal disease is defined as the presence of chronic kidney disease (CKD), acute kidney injury (AKI), or significant renal impairment diagnosed initially or during follow-up, indicated by reduced glomerular filtration rate (GFR), elevated serum creatinine, or other markers of renal dysfunction; ^e^autoimmune diseases include Ankylosing Spondylitis, Dermatomyositis, Polymyositis, Rheumatoid Arthritis, Psoriasis, Vasculitis, Sjögren’s Syndrome, and Systemic Lupus Erythematosus (SLE).

### Association of ANA and SSA with PFS of MM patients

**Figure 1:**
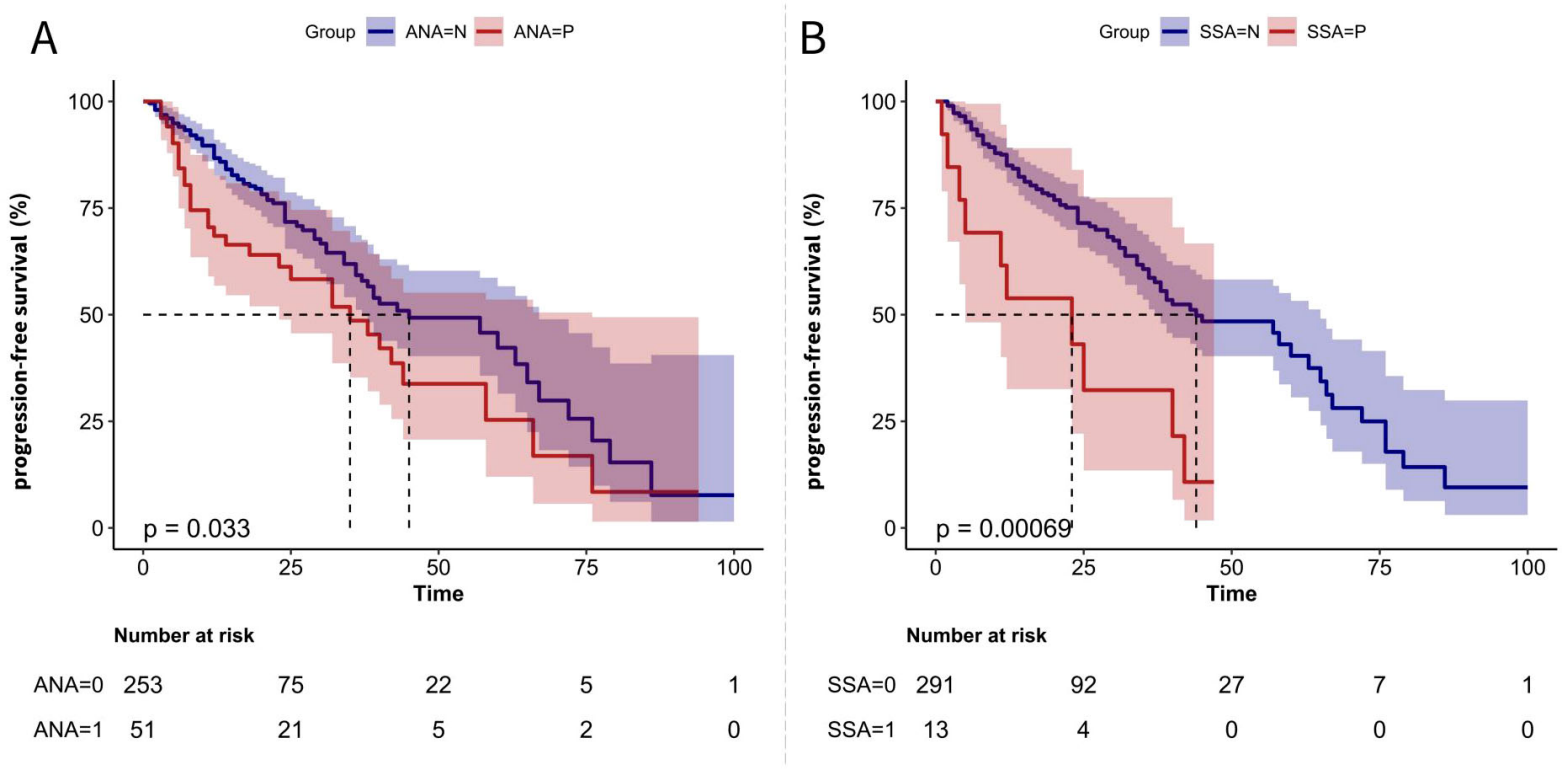
PFS Kaplan-Meier Curves for MM Patients Based on Autoantibody Status **(A)** Kaplan-Meier curve comparing PFS between ANA-negative (ANA=N) and ANA-positive (ANA=P) patients. **(B)** Kaplan-Meier curve comparing PFS between SSA-negative (SSA=N) and SSA-positive (SSA=P) patients. The p-values indicate the statistical significance of differences between the groups.

Kaplan–Meier analysis revealed that ANA-positive MM patients had a significantly shorter PFS compared to ANA-negative patients (**Figure 1A**). The median PFS for ANA-positive patients was 32 months, whereas for ANA-negative patients, it was 43 months. This trend was even more pronounced among SSA-positive patients (**Figure 1B**), where the median PFS for SSA-positive patients was 17 months compared to 44 months for negative.

**Table 2** presents the hazard ratios (HR) and 95% confidence intervals (CI) for PFS based on ANA and SSA status. In the crude model, ANA positivity was significantly associated with a higher risk of PFS (HR = 1.57, 95% CI 1.04-2.4, *p* = 0.034), while SSA positivity showed an even greater risk (HR = 2.93, 95% CI 1.53-5.64, *p* = 0.001). After adjusting for age, sex, DS stage, ISS, and R-ISS in Model 2, SSA positivity remained significantly associated with a higher risk of PFS (HR = 2.46, 95% CI 1.25-4.83, *p* = 0.009). In Model 3, which further adjusted for ASCT status, use of proteasome inhibitors, immunomodulators, daratumumab, monoclonal antibodies, and comorbidities, SSA-positive patients continued to exhibit a 112% higher risk of progression compared to negative (HR = 2.12, 95% CI 1.01-4.43, *p* = 0.047).

**Table 2:** Hazard ratios for progression-free survival based on ANA and SSA status across three cox regression models.

PFS	Events (incidence)^a^	Model 1^b^		Model 2^c^		Model 3^d^	
		HR (95%CI)	*P* value	HR (95%CI)	*P* value	HR (95%CI)	*P* value
ANA							
ANA-negative	85 (33.6)	1[Reference]		1[Reference]		1[Reference]	
ANA-positive	30 (58.8)	1.57 (1.04-2.4)	0.034	1.42 (0.91-2.21)	0.12	1.28 (0.79-2.09)	0.315
SSA							
SSA-negative	105 (36.1)	1[Reference]		1[Reference]		1[Reference]	
SSA-positive	10 (76.9)	2.93 (1.53-5.64)	0.001	2.46 (1.25-4.83)	0.009	2.12 (1.01-4.43)	0.047

PFS, Progression-Free Survival; HR, hazard ratios; CI, confdence interval; ANA, Antinuclear Antibodies; SSA, Anti-Sjögren’s-Syndrome-Related Antigen A.

a incidence: number of PFS per 100 person-months.

b Model 1: crude model.

c Model 2: adjusted by age, gender, DS stage, ISS stage, R-ISS stage.

d Model 3: adjusted for the variables in model 2 plus ASCT, Proteasome inhibitor, Immunomodulator, Daratumumab, Monoclonal Antibodies, Renal disease, Hypertension, Diabetes, Autoimmune disease.

### Association between treatment response and autoantibody status

A chi-square test was conducted to compare treatment response distributions between groups. The overall p-value for the ANA-positive and ANA-negative groups was 0.309. In contrast, the p-value for the SSA-positive and SSA-negative groups was 0.047, suggesting a statistically significant difference in treatment response distribution between SSA groups. Specifically, within the SD/PD category, the proportion of patients in the SSA-negative group was 36 (12.4%), whereas in the SSA-positive group, it was 5 (38.5%) (p = 0.023) (**Table 3**).

**Table 3:** Association between treatment response and autoantibody status.

IMWG Respones^a^, n(%)	Total (n=304)	ANA-Negative (n=253)	ANA-Postive (n=51)	p	SSA-Negative (n=291)	SSA-Postive (n=13)	p
				0.309			0.047
sCR/CR^b^	76 (25.0)	65 (25.7)	11 (21.6)	0.658	75 (25.8)	1 (7.7)	0.197
VGPR^c^	83 (27.3)	71 (28.1)	12 (23.5)	0.624	81 (27.8)	2 (15.4)	0.526
PR^d^	104 (34.2)	87 (34.4)	17 (33.3)	1	99 (34)	5 (38.5)	0.975
SD/PD^e^	41 (13.5)	30 (11.9)	11 (21.6)	0.104	36 (12.4)	5 (38.5)	0.023

a This table presents the distribution of treatment responses according to the International Myeloma Working Group (IMWG) criteria.

b sCR/CR (Stringent Complete Response/Complete Response): sCR requires the absence of clonal plasma cells in bone marrow, normal free light chain ratio, and negative immunofixation in serum and urine. CR is defined as negative immunofixation and <5% clonal plasma cells in bone marrow.

c VGPR (Very Good Partial Response): Defined as a ≥90% reduction in serum M-protein and urine M-protein <100 mg/24h.

d PR (Partial Response): Defined as a ≥50% reduction in serum M-protein and a ≥90% reduction in urine M-protein or urine M-protein <200 mg/24h.

e SD/PD (Stable Disease/Progressive Disease): SD indicates no significant change in M-protein levels, while PD is characterized by an increase of ≥25% in serum or urine M-protein levels, the development of new bone lesions, or a clinical progression of disease.

### Association Between FLCR and Autoantibody Status

The median FLCR values were 0.6 (0.5, 1.6) in the ANA-negative group and 0.6 (0.6, 2.9) in the ANA-positive group (p = 0.156), indicating no significant difference. Similarly, the χ² test for FLCR category distribution (0.01–100 vs. ≤0.01 or ≥100) showed no significant difference between ANA groups (p = 0.583).In contrast, the SSA-positive group had significantly higher FLCR values (1.8 [1.0, 78.9] vs. 0.6 [0.6, 1.6], p = 0.002). However, the proportion of patients with extreme FLCR values (≤0.01 or ≥100) did not significantly differ between SSA groups (7.7% vs. 15.5%, p = 0.7)(**Supplementary Table S3**).

### Subgroup analyses of the association between SSA and PFS

**Figure 2:**
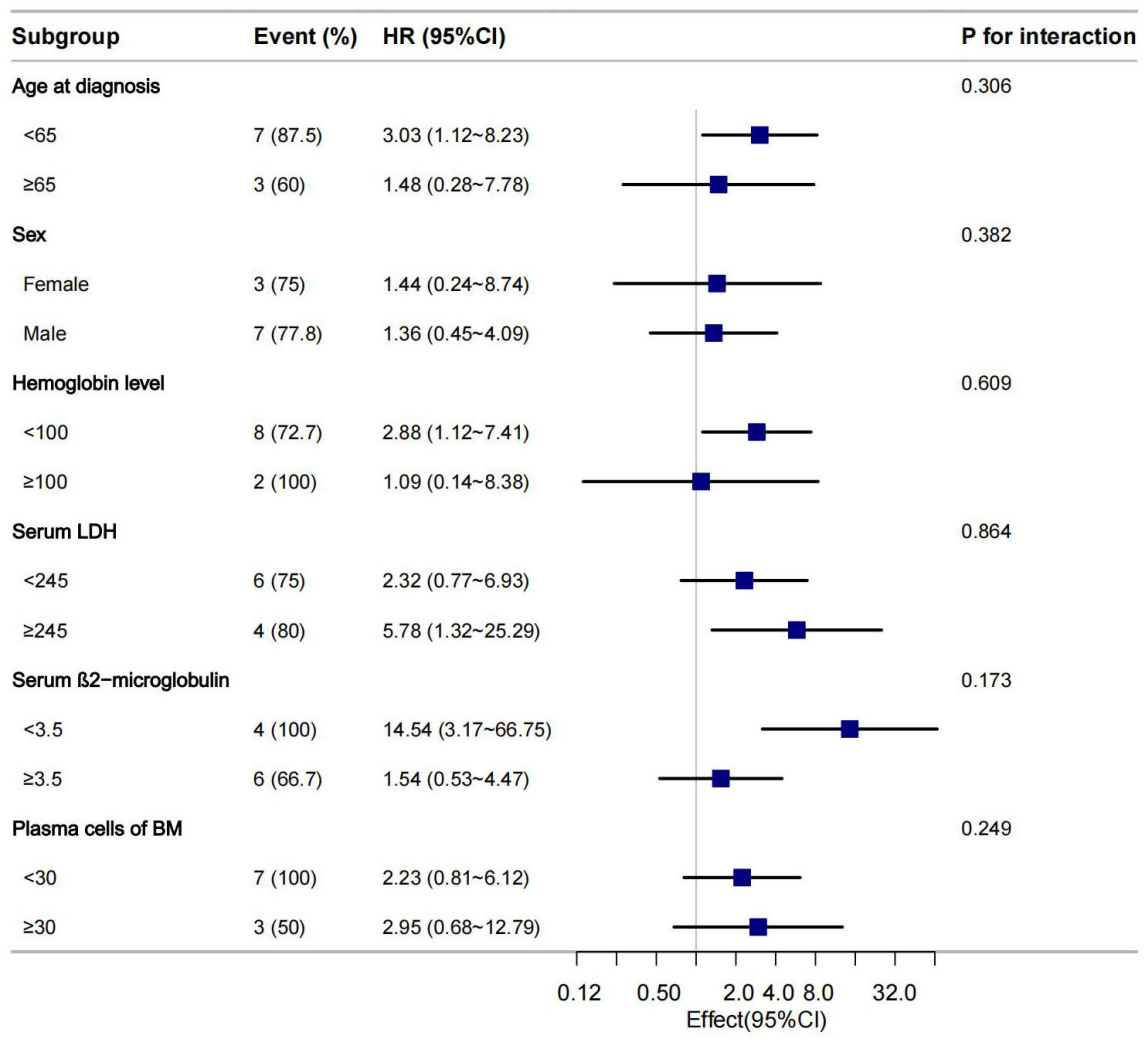
Subgroup analysis evaluating the relationship between SSA and PFS in MM.

We conducted subgroup analyses stratified by age, sex, Hb, LDH, serum β2M, and plasma cells of BM to further explore the association between SSA positivity and PFS. The results demonstrated a consistent effect of SSA positivity on PFS across all subgroups, with no evidence of significant interaction effects (**Figure 2**).

### Sensitivity analyses

The baseline characteristics after PSM are presented in **Supplementary Table S2**. The association between SSA positivity and PFS was evaluated using multiple models. After adjusting for the propensity score, this risk remained elevated (HR = 2.89, 95% CI: 1.27-6.57, p = 0.012). When PSM was applied, the HR increased to 3.66 (95% CI: 1.01-13.35, p = 0.049). Finally, inverse probability of treatment weighting (IPTW) yielded an even higher risk estimate (HR = 4.76, 95% CI: 1.76-12.9, p<0.001) (**Table 4**).

**Table 4:** Associations between SSA and PFS in the crude model and propensity score analyses.

Models	HR (95%CI)	*P* value
Crude model	2.93 (1.53-5.64)	0.001
^a^Adjusted for propensity score	2.89 (1.27-6.57)	0.012
^b^With matching	3.66 (1.01-13.35)	0.049
^c^With inverse probability weighting	4.76 (1.76-12.9)	<0.001

a Adjusted for propensity score, including all covariates.

b Results obtained from PSM using a 1:1 nearest neighbor algorithm with a caliper width of 0.2.

c Inverse probability of treatment weighting (IPTW) was applied, truncating weights at the 5th and 95th percentiles to address extreme values.

## Discussion

In our single-center, retrospective cohort study, we observed that ANA- and SSA-positive MM patients had significantly shorter PFS. This trend was more pronounced in SSA-positive patients, and Cox analysis further supported this observation, with SSA positivity remaining a significant risk factor in both propensity score-adjusted and weighted models.

Case reports, case series, and observational studies have previously reported an increased risk of MM in patients diagnosed with autoimmune diseases such as Sjögren’s syndrome, SLE, immune thrombocytopenia, and polymyositis (6, 10, 11, 26, 27). Although much research has explored the connection between autoimmune diseases and MM, few studies have examined the relationship between the biomarkers used to diagnose these autoimmune diseases, such as autoantibodies, and MM outcomes. A population-based Swedish study demonstrated that the increased MM risk in Sjögren’s syndrome patients was restricted to those positive for SSA and SSB autoantibodies (11). Our study builds on this by investigating the relationship between ANA, SSA, and MM progression, addressing a gap in the literature.

ANA is a group of autoantibodies targeting nuclear components, commonly found in various immune dysregulation disorders (28). SSA, a subtype of ANA, is closely associated with Sjögren’s syndrome and is also present in other autoimmune diseases (21). Our findings suggest that autoantibody positivity is associated with PFS in MM patients, offering a new perspective that might explain the conflicting results of previous observational studies. For example, while some studies have reported an increased MM risk in RA patients, others have found no such link (29, 30). We aimed to investigate the relationship between autoantibodies and MM by focusing on the shared feature of autoimmune diseases—the presence of autoantibodies, commonly used for diagnosis and monitoring. Compared to ANA, SSA demonstrated more robust results. One potential explanation is that SSA-positive patients may experience more profound immune dysfunction and immune activation (31). SSA antibodies primarily target ribonucleoprotein complexes, playing a role in B-cell activation and plasma cell differentiation (32). Studies have shown that immune activation, particularly through B-cell pathways, is critical in supporting MM progression, while ANA more broadly targets nuclear components without specifically affecting plasma cells (28). Thus, ANA-positive patients may exhibit a more generalized immune dysregulation, with less impact on MM progression.

Moreover, prior research suggests that the increased risk may be a consequence of treatment rather than the disease itself (6). For example, the use of corticosteroids has been linked to a higher MM risk (33). On the other hand, with the advent of the new therapeutic era in MM treatment, the use of proteasome inhibitors, immunomodulatory drugs, and monoclonal antibodies may influence autoantibody expression. Daratumumab, for instance, has been shown to significantly reduce autoantibody levels in SLE patients (34). In our study, we excluded patients who had undergone treatment before autoantibody testing, and the Cox regression models adjusted for treatment effects. The results remained robust, suggesting that SSA may play an independent role in MM prognosis.

Interestingly, although autoimmune diseases are more prevalent in women, we found that male patients had a higher prevalence of autoantibodies in our cohort, both for ANA and SSA (ANA: 68.4% vs. 31.6%; SSA: 69.2% vs. 30.8%). Among the 14 MM patients diagnosed with autoimmune disease in our study, 8 were male and 6 were female. However, subgroup analyses did not reveal any significant differences between sexes.

Our single-center study ensured high data quality with consistent diagnostic methods and comprehensive clinical data collection. This allowed for a more precise assessment of disease progression, which is often difficult in multicenter studies. However, we acknowledge the study’s limitations. Patients lost to follow-up were excluded, potentially introducing selection bias, and the small sample size for certain variables led to unstable HR and wide CI. For the same reason, we focused on two specific autoantibodies—ANA and SSA—in this study. Due to the limited sample size, we did not assess the association between autoantibodies and overall survival (OS). Therefore, our study highlights the need for future research to explore the relationship between autoantibodies and both PFS and OS in MM patients.

## Conclusion

In this study, we identified a significant association between SSA positivity and shorter progression-free survival (PFS) in multiple myeloma (MM) patients, with this effect persisting across various adjusted models and subgroup analyses. While our findings provide preliminary insights into the interplay between autoimmunity and MM progression, the intricate mechanisms underlying this relationship warrant further exploration. It is important to emphasize that the association between SSA positivity and shorter PFS identified in this study does not supersede validated prognostic factors such as the Revised International Staging System (R-ISS) or cytogenetic risk stratification. Instead, SSA may serve as a supplementary biomarker to refine risk assessment in specific subgroups, pending validation in larger cohorts.

## Data Availability

The raw data supporting the conclusions of this article will be made available by the authors, without undue reservation.
