# Supplementary material for: Association of ANA and SSA autoantibodies with progression-free survival in multiple myeloma: a retrospective cohort study
Source: Front Oncol. 2025 Feb 21;15:1529678. doi: 10.3389/fonc.2025.1529678 (PMC11885117; doi:10.3389/fonc.2025.1529678)
Supplement: Supplementary file 1 [file DataSheet1.docx]

**Supplementary Figure 1 Representative figures of immunofluorescence**


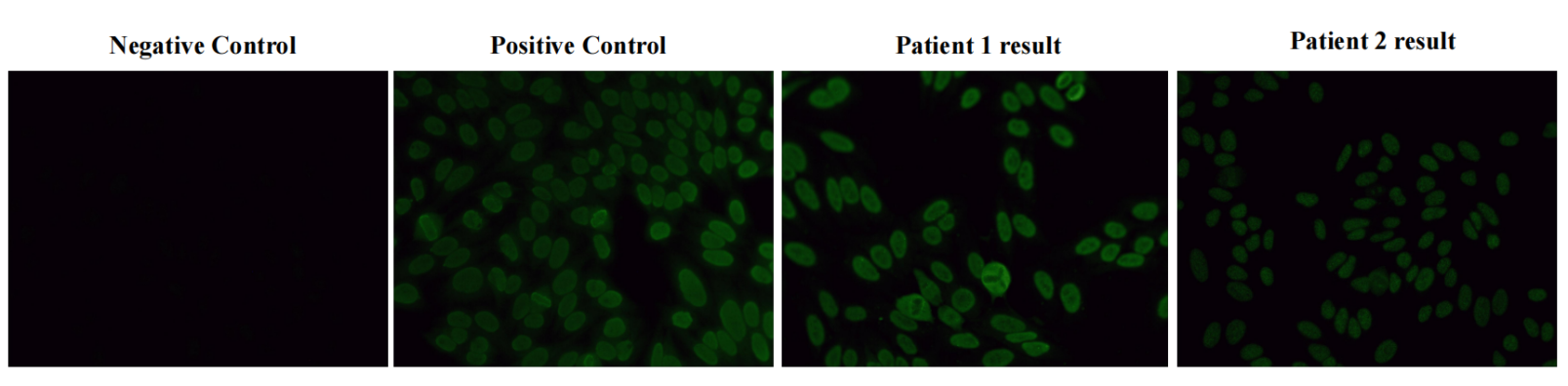


Representative figures of immunofluorescence (IF) using of HEp-2 cells staining were shown. Fixed HEp-2 substrate slides were incubated with internal quality controls (negative control and positive control) or patients serum followed by FITC-conjugated mouse anti-human IgG. Fluorescent figures were detected by fluorescence microscopy at 400 X magnification.

**Supplementary Figure 2 Distribution of Autoantibody Positivity Among multiple myeloma Patients**


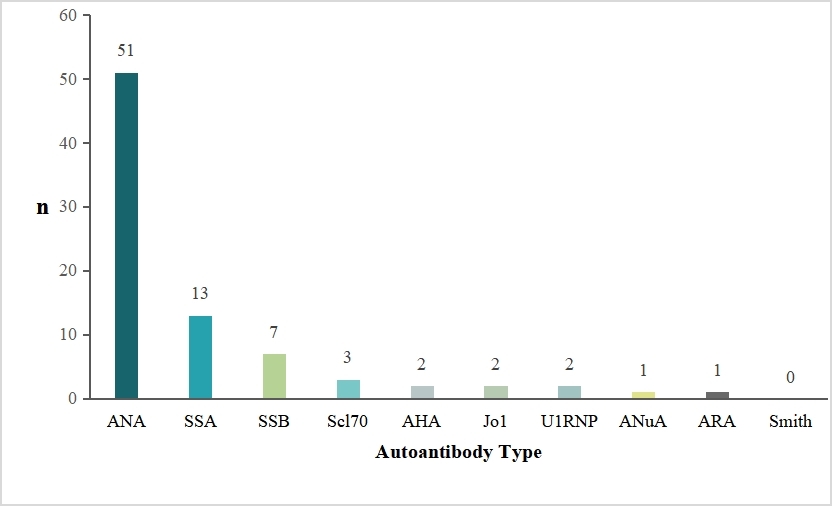


This figure shows the number of MM patients testing positive for various autoantibodies.

Abbreviations: ANA: Antinuclear Antibody; SSA: Anti-Sjögren's-Syndrome-Related Antigen A; SSB: Anti-Sjögren's-Syndrome-Related Antigen B; Scl70: Anti-topoisomerase I; AHA: Anti-histone antibody; Jo1: Anti-histidyl-tRNA synthetase antibody; U1RNP: Anti-U1 ribonucleoprotein; ANuA: Anti-nucleolar antibody; ARA: Anti-RNA antibody; Smith: Anti-Smith antibody.

**Supplementary Table 1 Baseline characteristics, and results in 14 MM patients diagnosed with autoimmune disease**

| **N** | **Age (years)/gender** | **Diagnosis(limited to autoimmune diseases)** | **ANA** | **SSA** |
| --- | --- | --- | --- | --- |
| 1 | 57/M | Ankylosing Spondylitis | - | - |
| 2 | 59/F | Dermatomyositis | - | - |
| 3 | 55/M | Polymyositis | - | - |
| 4 | 61/M | Rheumatoid Arthritis | + | - |
| 5 | 66/M | Psoriasis | - | - |
| 6 | 53/M | Ankylosing Spondylitis | - | - |
| 7 | 77/F | vasculitis | - | - |
| 8 | 59/F | Rheumatoid Arthritis | + | - |
| 9 | 48/M | Polymyositis | + | + |
| 10 | 64/F | Rheumatoid Arthritis | + | - |
| 11 | 78/M | Sjögren's Syndrome | + | + |
| 12 | 55/F | Rheumatoid Arthritis | + | - |
| 13 | 71/M | Rheumatoid Arthritis | + | - |
| 14 | 60/F | Systemic Lupus Erythematosus | + | - |

**Supplementary Table 2 Baseline characteristics of the study population after PSM matching**

| **Characteristics** | **SSA** | |  |  |
| --- | --- | --- | --- | --- |
|  | **Negative (n=13)** | **Positive (n=13)** | **SMD** | **SMD 0.1** |
| Age ≥65 | 6 (46.2) | 5 (38.5) | 0.156 | >0.1 |
| Male | 10 (76.9) | 9 (69.2) | 0.174 | >0.1 |
| M protein type, n (%) | - | - | 0.213 | >0.1 |
| Lambdad Light chain | 10 (76.9) | 8 (61.5) | 0.338 | >0.1 |
| Hemoglobin level ≥100 | 3 (23.1) | 2 (15.4) | 0.196 | >0.1 |
| Serum β2-microglobulin ≥3.5 | 10 (76.9) | 9 (69.2) | 0.174 | >0.1 |
| Serum LDH ≥245 | 4 (30.8) | 5 (38.5) | 0.162 | >0.1 |
| Serum calcium level ≥2.65 | - | - | <0.001 | >0.1 |
| Serum creatinine level ≥177 | 4 (30.8) | 3 (23.1) | 0.174 | <0.1 |
| Plasma cells of BM ≥30 | 5 (38.5) | 6 (46.2) | 0.156 | >0.1 |
| High risk cytogenetic abnormalitie, n (%)^b^ | 11 (84.6) | 11 (84.6) | <0.001 | >0.1 |
| DS stage, n (%) | - | - | <0.001 | <0.1 |
| ISS stage, n (%) | - | - | <0.001 | <0.1 |
| R-ISS stage, n (%) | - | - | 0.168 | >0.1 |
| ASCT-Yes | 0 (0) | 1 (7.7) | 0.408 | >0.1 |
| Proteasome inhibitor-Yes | 10 (76.9) | 10 (76.9) | <0.001 | <0.1 |
| Immunomodulator-Yes | 9 (69.2) | 9 (69.2) | <0.001 | <0.1 |
| Monoclonal Antibodies-Yes |  |  | <0.001 | <0.1 |
| Renal disease-Yes | 2 (15.4) | 2 (15.4) | <0.001 | <0.1 |
| Hypertension-Yes | 3 (23.1) | 2 (15.4) | 0.196 | >0.1 |
| Diabetes-Yes | 13 (100) | 13 (100) | <0.001 | <0.1 |
| Autoimmune disease-Yes | 2 (15.4) | 2 (15.4) | <0.001 | <0.1 |

**Supplementary Table 3 Association Between FLCR and Autoantibody Status**

| **Variables** | **Total**  **（n=304）** | **ANA-Negative**  **（n=253）** | **ANA-Postive**  **（n=51）** | **p** | **SSA-Negative**  **（n=291）** | **SSA-Postive**  **（n=13）** | **p** |
| --- | --- | --- | --- | --- | --- | --- | --- |
| **FLCR，Median** | **0.6 (0.6, 1.7)** | **0.6 (0.5, 1.6)** | **0.6 (0.6, 2.9)** | **0.156** | **0.6 (0.6, 1.6)** | **1.8 (1.0, 78.9)** | **0.002** |
| **FLCR** |  |  |  | **0.583** |  |  | **0.7** |
| **0.01–100** | **258 (84.9)** | **216 (85.4)** | **42 (82.4)** |  | **246 (84.5)** | **12 (92.3)** |  |
| **≤0.01，≥100** | **46 (15.1)** | **37 (14.6)** | **9 (17.6)** |  | **45 (15.5)** | **1 (7.7)** |  |
